# Circular RNA hsa_circ_0000848 Promotes Trophoblast Cell Migration and Invasion and Inhibits Cell Apoptosis by Sponging hsa-miR-6768-5p

**DOI:** 10.3389/fcell.2020.00278

**Published:** 2020-05-19

**Authors:** Hui Wang, Jianming Zhang, Zhiyong Xu, Jingxin Yang, Yong Xu, Yang Liu, Bohong Li, Jiansheng Xie, Jing Li

**Affiliations:** ^1^Department of Obstetrics and Gynecology, Shenzhen Maternity and Child Healthcare Hospital, Southern Medical University, Shenzhen, China; ^2^Medical Genetic Center, Shenzhen Maternity and Child Healthcare Hospital, Southern Medical University, Shenzhen, China; ^3^Department of Obstetrics and Gynecology, Nanfang Hospital, Southern Medical University, Guangzhou, China

**Keywords:** fetal growth restriction, circ_0000848, hsa-miR-6768-5p, ceRNA, human extravillous trophoblast cell line

## Abstract

**Background:**

Fetal growth restriction (FGR) is a worldwide problem, and a major cause of perinatal morbidity. The precise molecular mechanisms involved in placental development and function during FGR remain poorly understood. Circular RNAs (circRNAs) are important biological molecules associated with disease pathogenesis. However, the role of circRNAs in FGR has not been well studied.

**Methods:**

circRNA expression profiles in placental tissues with and without FGR were identified by circRNA microarray. circRNA expression was verified by quantitative reverse-transcription PCR (RT-qPCR) assay. The effect of hsa_circ_0000848 and hsa-miR-6768-5p on HTR-8 cell apoptosis, migration, and invasion was evaluated. The association between hsa_circ_0000848 and hsa-miR-6768-5p was confirmed by dual luciferase activity and anti-AGO2 RNA immunoprecipitation (RIP) assays. Protein levels were examined via western blotting.

**Results:**

RT-qPCR results showed that hsa_circ_0000848 expression was significantly down-regulated in FGR placenta. Hsa_circ_0000848 overexpression and hsa-miR-6768-5p inhibitor suppressed apoptosis, and promoted cell migration and invasion. In addition, hsa_circ_0000848 overexpression and hsa-miR-6768-5p inhibitor increased the protein abundance of BCL2, MMP2 and MMP9, and decreased the protein abundance of cleaved caspase-3, cleaved caspase-9, and BAX, whereas hsa_circ_0000848 knockdown caused the opposite effect. Moreover, a significant increase in hsa-miR-6768-5p expression and a negative correlation between hsa_circ_0000848 and hsa-miR-6768-5p were identified in the FGR tissues. Luciferase reporter and RIP assay results revealed binding of hsa-miR-6768-5p to hsa_circ_0000848. Furthermore, hsa-miR-6768-5p overexpression eliminated the effect of hsa_circ_0000848 overexpression in HTR-8 cells.

**Conclusions:**

hsa_circ_0000848 expression is significantly down-regulated in the FGR placenta. hsa_circ_0000848 promotes trophoblast cell migration and invasion, and inhibits cell apoptosis via the sponging of hsa-miR-6768-5p. Our study provided a novel insight into mechanisms underlying the pathogenesis of FGR, as well as into new strategies for the treatment of FGR.

## Introduction

Fetal growth restriction (FGR), also known as intrauterine growth restriction (IUGR), is defined as a fetal birth weight less than 2,500 g after 37 weeks gestation, a fetal weight less than two standard deviations below the mean weight for the same gestational age, or a fetal weight below the 10th percentile of the normal weight for the same gestational age (Sharma et al., 2016a; Figueras and Gratacos, 2017; Nardozza et al., 2017). FGR is a worldwide problem, and the major cause of multifarious mortality and morbidity in the neonatal and fetal population (Sharma et al., 2016b; Nardozza et al., 2017). FGR affects 5–10% of pregnancies, and the stillbirth rate of FGR is approximately 30% (Gynecologists, 2013; Nardozza et al., 2017). The etiology and pathogenesis of FGR is complex; maternal, fetal, and uteroplacental factors are involved (Gynecologists, 2013; Nardozza et al., 2017). Increases in inflammatory cytokine levels in maternal plasma can cause FGR (Robb et al., 2017). In addition, placental factors are widely responsible for FGR, and poor placental perfusion is the main cause of FGR (Nardozza et al., 2017). Inappropriate invasion of trophoblast cells may lead to poor placental perfusion (Mifsud and Sebire, 2014; Deng et al., 2015). During normal pregnancy, trophoblast cells invade maternal decidua, rapidly proliferate and differentiate, and intrude into the endometrium matrix in a controlled manner. Excessive apoptosis of trophoblast cells is one of the factors leading to deficient trophoblast invasion (Reister et al., 2001; Huppertz et al., 2005). At present, the precise molecular mechanisms regulating trophoblast cells in FGR are poorly understood. Therefore, identification of potential molecular FGR markers in the placenta, and investigating their roles in both the regulation of inappropriate invasion and the excessive apoptosis of trophoblast cells, is essential to fully understand the etiology and pathogenesis of FGR.

Circular RNAs (circRNAs) are ubiquitously present in mammalian cells, and are involved in gene expression regulation at the transcriptional and post-transcriptional levels (Salzman, 2016; Hsiao et al., 2017). CircRNAs can be formed by circularization of one or more exons, both exons and intron sequences, or intronic sequences by a process known as “back-splicing” (Lasda and Parker, 2014). circRNAs have a closed covalent ring structure, so they are resistant to RNA exonuclease and are more stable than linear RNAs and microRNAs (miRNAs) (Qu et al., 2015). Many studies have shown that circRNAs can bind to miRNAs and function as “miRNA sponges” to regulate gene transcription (Hansen et al., 2013; Liu et al., 2019). Accumulating evidence supports that circRNAs are important biological molecules associated with disease pathogenesis (Kristensen et al., 2019; Yu et al., 2019). Shen et al. (2018) identified 44 differentially expressed circRNAs in pig livers using adult IUGR and normal pigs as models, via RNA sequencing. They identified ATF4/miR-214/circRNA7964 and TCF7/miR-22-3p/circRNA16347 as two competing endogenous networks by bioinformatic analysis (Shen et al., 2018). In addition, circPAPPA and circTRNC18 play regulatory roles in trophoblast cells (Shen et al., 2019; Zhou et al., 2019) and a subset of placental cells, thus performing critical functions in fetal growth and survival. These results indicate that circRNAs may play key regulatory roles in FGR. However, the role of circRNAs in FGR has not been well studied.

In this study, we first performed microarray analysis to investigate the expression profiles of circRNAs in FGR tissues. Furthermore, we investigated the function and regulatory mechanism of hsa_circ_0000848 in HTR-8 cells, a human extravillous trophoblast (EVT) cell line, to study trophoblast functions including cell fusion, migration, and invasion. This study provided novel insights into the mechanism of the pathogenesis of FGR, as well as into new strategies for FGR treatment.

## Materials and Methods

### Tissue Samples

Forty pregnant women with FGR were included in this study, and their detailed characteristics are shown in Supplementary Table S1. FGR was defined as an estimated fetal weight of below the 10th percentile for gestational age, calculated using the Hadlock equation, and confirmed at birth by a neonatal weight below the 10th percentile. Forty gestation-matched placentas from healthy pregnant woman were used as controls, and their detailed characteristics are shown in Supplementary Table S1. All participants had normal platelet counts, normal functioning of the livers and kidneys, normal blood pressure, and normal blood sugar concentration. The pregnant women of both groups were single pregnancy, and there were no other accompanying complications during pregnancy. The collected placenta was divided into four quadrants, and samples from each quadrant were combined for circRNA microarray analysis and RT-qPCR assay. Placental tissues were placed in RNA-later solution (Sangon Biotech, Shanghai, China) immediately after delivery, then frozen in liquid nitrogen. This study was approved by the Ethics Committee of Shenzhen Maternity and Child Health Hospital, Shenzhen, China. All participants provided written informed consent.

### CircRNA Microarray Analysis

Six samples (three samples each from FGR tissues and healthy controls) from the first individuals enrolled in this study, were used in the microarray screening. Sample preparation and microarray hybridization were performed according to the standard protocols of Arraystar Circular RNA Microarray Version 2.0 (Arraystar, Rockville, MD, United States). After washing, arrays were scanned with the Agilent G2505C Scanner (Agilent Technologies, Inc., Santa Clara, CA, United States). Agilent Feature Extraction software (version 11.0.1.1) was used to extract raw data. Quantile normalization was conducted using customized R software package. circRNAs having fold changes >1.5 and *p*-values <0.05 were determined as being significantly differentially expressed. CircRNA/microRNA interactions were predicted using Arraystar’s home-made miRNA target prediction software. Microarray analysis was performed by the KangChen Bio-tech company (Shanghai, China).

### Cell Culture and Transfection

HTR-8/SVneo cells were obtained from the American Type Culture Collection (ATCC, Manassas, VA, United States), and cultured in RPMI 1640 medium (Gibco, Carlsbad, CA, United States), supplemented with 10% fetal bovine serum (Gibco), 1% penicillin/streptomycin, and 1% L-glutamine at 37°C, in humidified air with 5% CO_2_.

Full length hsa_circ_0000848 (position: chr18:46470601-46470865; spliced length: 264 nt) was cloned into the pLCD5H-ciR plasmid between *Eco*RI and *Bam*HI sites (Guangzhou Geneseed Biotech Co., Ltd., China), by DNA synthesis *in vitro*, to construct the hsa_circ_0000848 overexpression vector (ov-circ_0000848). Empty pLCD5H-ciR plasmid was used as a negative control (NC). Small interfering RNA (siRNA) targeting hsa_circ_0000848 (si-circ_0000848; sense sequence: AAUGUGGGGGUGGAGGCUUTT), negative control siRNA (si-NC; sense sequence: UUCUCCG AACGUGUCACGUTT), hsa-miR-6768-5p mimic (CACAC AGGAAAAGCGGGGCCCUG), hsa-miR-6768-5p inhibitor (CA GGGCCCCGCUUUUCCUGUGUG), negative control miRNA inhibitor (miR-NC inhibitor; UCUACUCUU UCUAGGAGGUUGUGA), and negative control miRNA (miR-NC; UCACAACCUCCUAGAAAGAGUAGA) were obtained from GenePharma Co., Ltd. (Shanghai, China).

HTR-8 cells (2 × 10^5^ cells/well) were seeded in six-well plates and grown overnight. On the following day, cells were washed, placed in serum-free medium, then transfected with the above vector (1 μg/per well), siRNAs (final concentration, 50 nM), and miRNAs (final concentration, 50 nM) according to the manufacturer’s protocol using Lipofectamine^TM^ 2000 Reagent (Invitrogen, Carlsbad, CA, United States). After 6 h, the medium was replaced with complete medium, and cells were cultured at 37°C, in humidified air with 5% CO_2_. After transfection for 48 h, cells were harvested for the following assays, except for the cell migration and invasion assays.

### RNA Isolation and Quantitative Reverse-Transcription PCR (RT-qPCR) Assay

Total RNA was extracted from tissue samples or cells using TRIzol reagent (Invitrogen). Total RNAs were quantified using a NanoDrop ND-1000 spectrophotometer. After incubation for 15 min at 37°C with 3 U/μg of RNase R (Epicentre Biotechnologies, Madison, WI, United States), the RNA was used to obtain cDNA using the ImProm-IITM Reverse Transcription System (Promega, Madison, WI, United States). Random primers were used for reverse transcription to detect circRNAs. To detect miRNA expression, the primers used for reverse transcription and PCR detection were designed based on the stem-loop principle. Quantitative RT-PCR (RT-qPCR) analysis was performed using SYBR GREEN qPCR Super Mix (Promega, United States). The primers used for RT-qPCR are shown in Supplementary Table S2. GAPDH was used as an internal control for circRNAs and mRNAs. U6 was used as an internal control for miRNAs. The RT-qPCR assay was performed using three independent replicates. Relative expression level of RNA was calculated using the 2^–^^Δ^^Δ^^*Ct*^ method, in which one sample from the FGR or normal group was randomly chosen and set to 1.

### Bioinformatic Analysis

The detailed information of circRNAs was obtained from circBase. Target miRNAs of hsa_circ_0000848 were predicted using the TargetScan and miRanda databases. The target mRNAs of hsa-miR-6768-5p were predicted using MirAncesTar, miRDB, Mirza-G, and RNAhybrid. The signaling pathways and biological processes of hsa-miR-6768-5p targets were analyzed using the KEGG (Kyoto Encyclopedia of Genes and Genomes) or GO (Gene Ontology) databases.

### Dual Luciferase Activity Assay

For the construction of the recombinant luciferase reporter plasmid, fragments of wild-type hsa_circ_0000848 containing the binding sequences of hsa-miR-6768-5p were amplified and cloned into the psi-CHECK-2 vector (Promega) and termed ‘wt-circ_0000848’. The binding sequences of hsa-miR-6768-5p in the recombinant plasmids were mutated via site-directed mutagenesis using one-step overlap extension PCR and termed “mut-circ_0000848.” HTR-8 cells were plated on 24-well plates, and co-transfected with 100 ng recombinant plasmid and 50 nM of hsa-miR-6768-5p mimic or miR-NC. Forty-eight hours after transfection, Firefly and Renilla luciferase activities were measured using the Dual-Luciferase Reporter Assay System (Promega), according to the manufacturer’s instructions. Relative luciferase activity was calculated using the ratio of Renilla and Firefly luciferase activities (R/F). Three independent experiments were performed.

### Anti-AGO2 RNA Immunoprecipitation (RIP) Assay

The RIP assay was carried out according to the Magna RIP RNA-Binding Protein Immunoprecipitation Kit instructions (Millipore, Bedford, MA, United States). Briefly, approximately 1 × 10^7^ cells, transfected with hsa-miR-6768-5p mimic or miR-NC, were lysed in polysome lysis buffer including protease inhibitors. Before immunoprecipitation, partial cell lysate was collected for use as a positive control for the RIP assay, termed “input.” Subsequently, 100 μl of cell lysate was incubated with magnetic bead-antibody (IgG or Ago2) complex at 4°C, overnight, to pulldown the complex that binds to hsa-miR-6768-5p mimic or miR-NC. RNA in the cell lysate of the positive control and RIP products was extracted and purified. The levels of hsa_circ_0000848 in purified RNA were detected by RT-qPCR. All assays were performed using three independent replicates.

### Cell Migration and Invasion Assay

After transfection for 24 h, HTR-8 cells were starved for 24 h and subsequently suspended in serum-free medium. Transwell chambers (Corning, Steuben County, NY, United States) with 24 wells and 8 μm pores were coated with fibronectin for the migration assay, or with Matrigel (BD Biosciences, Bedford, MA, United States) for the invasion assay. HTR-8 cells were seeded in the upper chambers. After 24 h of incubation at 37°C, cells that had successfully migrated onto the lower membrane surface, or had successfully invaded through the membrane, were fixed in 4% formaldehyde and stained with 0.5% Crystal Violet. The number of migrating or invading cells was counted using a light microscope (Nikon company, Tokyo, Japan). All assays were performed using three independent replicates.

### Cell Apoptosis Experiment

The apoptosis assay was performed using the Annexin V-FITC/PI Apoptosis Detection Kit (KeyGEN Biotech, Nanjing, China). Briefly, 2 × 10^5^ cells/well were seeded into six-well plates. Forty-eight hours after transfection, cells were harvested and washed with cold PBS. Then, cells were incubated with 5 μl Annexin V-FITC and 5 μl propidium iodide (PI) on ice for 5 min in the dark. Apoptosis of HTR-8 cells was analyzed using a BD FACSCalibur flow cytometer following the manufacturer’s instructions. Cell apoptosis data were interpreted using the FlowJo software version 8 (FlowJo, Ashland, OR, United States). The percentages of early (Annexin V-FITC^+^/PI^–^) and late (Annexin V-FITC^+^/PI^+^) apoptotic cells (as calculated by the number of early or late cells over the total number of all apoptotic cells) were analyzed to evaluate the effect on apoptosis. All assays were performed using three independent replicates.

### Western Blot Analysis

Total proteins were extracted using the RIP assay strong lysis buffer (Beyotime, Shanghai, China). Protein concentrations were quantified using the Bio-Rad Protein Assay Kit (Bio-Rad Laboratories, Hercules, CA, United States). Sodium dodecyl sulfate-polyacrylamide gel electrophoresis was then performed to separate the proteins, which were then transferred onto a polyvinylidene fluoride membrane. Then, the membranes were incubated with primary antibodies (BLC2, 1:1,000, ab32124; MMP2, 1:2,000, ab215986; MMP9, 1:1,000, ab219372; caspase-3, 1:1,500, ab32351; caspase-9, 1:500, ab2324; and BAX, 1:1,000, ab199677; all purchased from Abcam, Cambridge, MA, United States) overnight at 4°C. Afterward, the membranes were washed and incubated with a secondary antibody (anti-rabbit IgG, HRP-linked Antibody, #7074, 1:5,000; Cell Signaling Technology, Beverly, MA, United States) at room temperature for 2 h. Finally, the bands on the membranes were visualized using enhanced chemiluminescence (Pierce, Rockford, IL, United States), and signals were exposed to X-ray. All films were scanned and densitometric analysis was performed three times using Image Pro-Plus 6.0 software (Media Cybernetics, Silver Spring, MD, United States). The relative protein abundance was expressed as the ratio of the target protein densitometric value to the GAPDH densitometric value.

### Statistical Analysis

Statistical analyses were performed using SPSS version 18.0 (IBM Corp, Armonk, NY, United States) and GraphPad Prism version 6.0 (GraphPad Software, San Diego, CA, United States). Data were expressed as mean ± standard deviation (SD) based on three separate experiments. Differences between the two experimental groups were analyzed using unpaired *t*-tests when the data was normally distributed, or non-parametric tests when the data was not normally distributed. The correlation between hsa_circ_0000848 and hsa-miR-6768-5p in the FGR placental tissues was analyzed by linear regression analysis using GraphPad Prism version 7.0 (GraphPad Software). *P* < 0.05 was considered statistically significant.

## Results

### Characteristics of the Participants

As shown in Supplementary Table S1, the average maternal ages in the FGR and normal groups were 30.58 ± 5.41 years, and 30.53 ± 4.62 years, respectively, and were not significantly different between the groups (*P* = 0.96). The average gestation times in the FGR and normal groups were 37.77 ± 1.6 weeks, and 38.46 ± 0.88 weeks, respectively, which were significantly different (*P* = 0.0156). The birth weights in the FGR and normal groups were 2,231 ± 352 g, and 3,385.75 ± 429 g, respectively, and were significantly differenct (*P* < 0.0001).

### Identification of Differentially Expressed circRNAs in FGR Placenta

**FIGURE 1:**
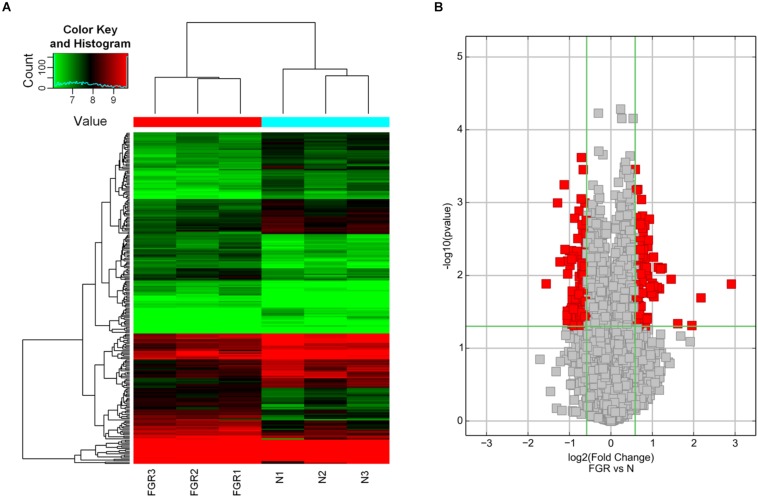
Bioinformatic analysis of the results of circRNA microarray analysis. **(A)** Hierarchical clustering of differentially expressed circRNAs in FGR vs Normal samples (N). The results of hierarchical clustering show a distinguishable circRNA expression profile among samples. Black: no change; red: upregulation; green: down-regulation. **(B)** Volcano plots of differentially expressed circRNAs in FGR vs Normal samples (N). Volcano plots are useful tools for visualizing differential expression between two different conditions. The red point in the plot represents the statistically significant differentially expressed circRNAs.

To study the expression pattern of circRNAs in FGR and normal placental tissues, we used a circRNA microarray to identify differentially expressed circRNAs. Hierarchical clustering distinguished circRNA expression levels in the associated heat map (Figure 1A). The red points in the Volcano plots represent the statistically significantly differentially expressed circRNAs between the FGR and normal groups based on statistical criteria of fold-change >1.5 and *P* < 0.05 (Figure 1B). Based on our statistical criteria, 244 circRNAs were found to be differentially expressed between the placental tissues of the FGR and control groups. For high stringency, the definition for a differentially regulated gene also requires FDR < 0.05. FDR, fully named as False Discovery Rate, is a compromising point of view regarding the error in multiple comparisons suggested by Benjamini and Hochberg (Benjamini et al., 2001). In our circRNA microarray data, the FDRs of the differentially expressed circRNAs are >0.05. Nevertheless, the results of our microarray analysis were used for further characterization of differentially expressed circRNA based on our initial statistical criteria.

### Hsa_circ_0000848 Expression Was Significantly Down-Regulated in FGR Placenta

**FIGURE 2:**
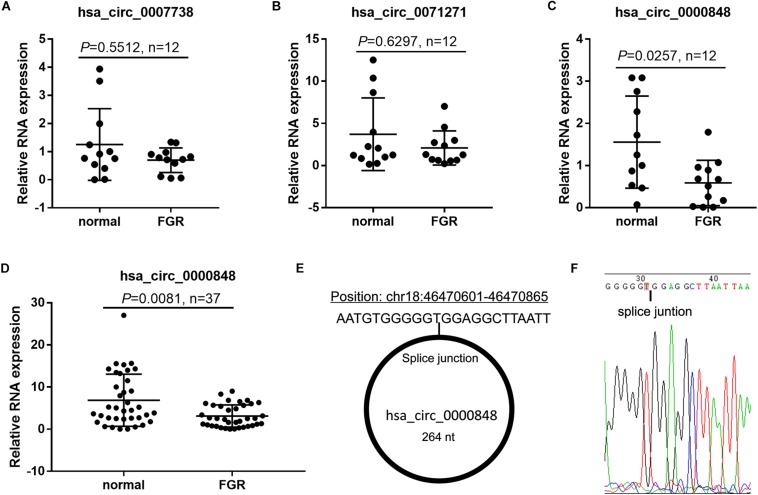
Hsa_circ_0000848 expression was significantly down-regulated in FGR placenta. **(A–C)** Expression levels of hsa_circ_0007738, hsa_circ_0071271, and hsa_circ_0000848 in FGR and normal placental tissues (*n* = 12). **(D)** Expression levels of hsa_circ_0000848 in FGR and normal placental tissues (37). **(E)** Detailed information of hsa_circ_0000848 from circBase. **(F)** Sequencing analysis around the splice junction of hsa_circ_0000848 fragment in RT-qPCR. FGR vs normal using a non-parametric test, n = 37.

To verify circRNA expression in the FGR placenta, we chose hsa_circ_0007738, hsa_circ_0071271, and hsa_circ_0000848, which had the greatest expression differences between the FGR and normal groups (fold-change >1.5 and *P* < 0.05), for further analysis. Hsa_circ_0000848 expression level was significantly lower in 12 FGR placental tissues than in 12 normal placenta tissues (*P* = 0.0257), whereas hsa_circ_0007738 (*P* = 0.5512) and hsa_circ_0071271 (*P* = 0.6297) expression levels did not change significantly (Figures 2A–C). Subsequently, hsa_circ_0000848 expression was further verified in FGR and normal placenta tissues (*n* = 37 each) and was found to be significantly lower in FGR tissues than in normal placenta tissues (*P* = 0.0081) (Figure 2D). Detailed information regarding hsa_circ_0000848, found in circBase, is shown in Figure 2E. The position of the splice junctions shown in circBase can be verified by our sequence analysis of the hsa_circ_0000848 fragment in RT-qPCR (Figure 2F).

### Hsa_circ_0000848 Inhibited Apoptosis and Promoted Migration and Invasion of HTR-8 Cells

**FIGURE 3:**
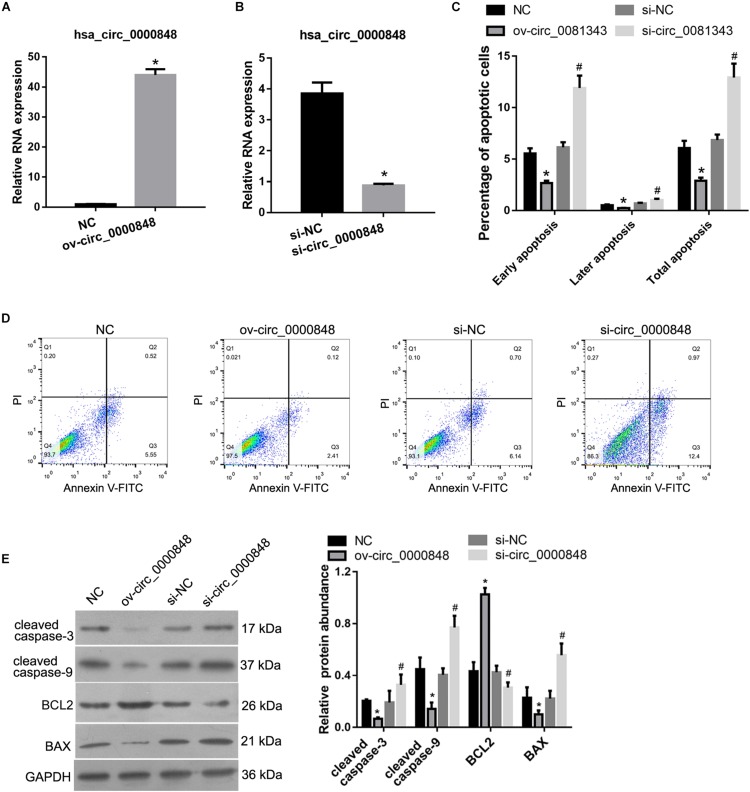
Effect of hsa_circ_0000848 overexpression or knockdown on apoptosis of HTR-8 cells. **(A)** hsa_circ_0000848 is overexpressed in HTR-8 cells after transfection with hsa_circ_0000848 containing plasmid (ov-circ_0000848) compared to negative control plasmid (NC). *P < 0.05, when compared to NC using unpaired *t*-tests. **(B)** hsa_circ_0000848 expression is lower in HTR-8 cells after transfection with siRNA targeting hsa_circ_0000848 (si-circ_0000848) compared to that in NC siRNA (si-NC). *P < 0.05, when compared to si-NC using unpaired *t*-tests. **(C,D)** Effect of hsa_circ_0000848 overexpression or knockdown on the apoptosis of HTR-8 cells. Panel **C** shows quantification of the percentage of apoptotic cells. Panel **D** shows representative graphs of the apoptosis detected using a flow cytometer. **(E)** Effect of hsa_circ_0000848 overexpression or knockdown on protein abundance of MMP-2 and MMP-9 in HTR-8 cells. The left panel includes representative images of western blots. The right panel graphs represent the quantification of relative protein abundance. *P < 0.05, when compared to NC using unpaired *t*-tests. ^#^*P* < 0.05, when compared to si-NC using unpaired *t*-tests.

To evaluate the functional role of hsa_circ_0000848 in FGR, the HTR-8 cell line was studied. As shown in Figure 3A, hsa_circ_0000848 expression levels in HTR-8 cells of the ov-circ_0000848 group were 44-fold higher than those of the NC group, indicating that hsa_circ_0000848 was successfully overexpressed in HTR-8 cells. As shown in Figure 3B, hsa_circ_0000848 expression levels in HTR-8 cells of the si-circ_0000848 group were 77% lower than those of the si-NC group, indicating that hsa_circ_0000848 was successfully knocked down in HTR-8 cells.

We subsequently characterized the role of hsa_circ_0000848 in HTR-8 cell apoptosis. As shown in Figures 3C,D, the percentage of apoptotic cells in the ov-circ_0000848 group was clearly lower than that in the NC group, and the percentage of apoptotic cells in the si-circ_0000848 group was clearly more than that in the si-NC group. In addition, the abundance of the apoptosis-related proteins cleaved caspase-3, cleaved caspase-9, BCL2, and BAX was also determined. The abundance of BCL2 was higher in the ov-circ_0000848 group than in the NC group, and was lower in the si-circ_0000848 group than in the si-NC group (Figure 3E). Moreover, the abundance of cleaved caspase-3, cleaved caspase-9, and BAX was lower in the ov-circ_0000848 group than in the NC group, and was higher in the si-circ_0000848 group than in the si-NC group (Figure 3E).

**FIGURE 4:**
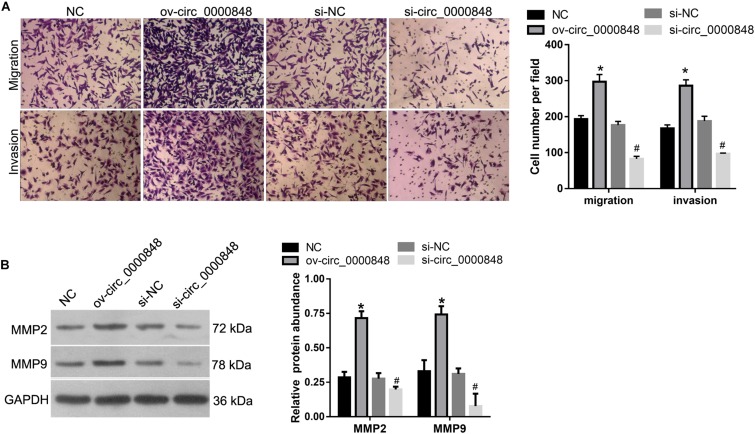
Effect of hsa_circ_0000848 overexpression or knockdown on migration and invasion of HTR-8 cells. **(A)** The results of transwell assay. The left panel includes representative images of migration and invasion by Transwell assay. The right panel includes bar graphs representing the quantification of migrated or invaded cell numbers. **(B)** Effect of hsa_circ_0000848 overexpression or knockdown on protein abundance of cleaved caspase-3, cleaved caspase-9, BCL2, and BAX in HTR-8 cells. The left panel includes representative images of western blots. The right panel graphs denote the quantification of relative protein expression levels. *P < 0.05, when compared to NC using unpaired *t*-tests. ^#^*P* < 0.05, when compared to si-NC using unpaired *t*-tests.

Next, we investigated the role of hsa_circ_0000848 in the migration and invasion of HTR-8 cells by performing a Transwell assay. The results showed that the number of cells that migrated to, or invaded, the bottom chamber was higher in the ov-circ_0000848 group than in the NC group, and was lower in the si-circ_0000848 group than in the si-NC group (Figure 4A). In addition, the abundance of metastasis-related proteins MMP-2 and MMP-9 was also determined. The abundance of MMP-2 and MMP-9 was higher in the ov-circ_0000848 group than in the NC group, and was lower in the si-circ_0000848 group than in the si-NC group (Figure 4B).

### Hsa_circ_0000848 Directly Targeted hsa-miR-6768-5p

We identified 23 microRNA response elements (MREs) within the sequence of hsa_circ_0000848 using the TargetScan, miRanda, and RNAhybid tools, as shown in Supplementary Figure S1. Total context^++^ score (Agarwal et al., 2015) was used for prioritizing the potential targets of hsa_circ_0000848. Among these miRNAs shown in Supplementary Figure S1, the context^++^ scores of hsa-miR-609, hsa-miR-514b-5p, hsa-miR-6768-5p, hsa-miR-6873-5p, hsa-miR-4330, hsa-miR-4267, and hsa-miR-6764-5p were higher than those of the other miRNAs. Therefore, the seven miRNAs above were chosen to further identify the direct target.

**FIGURE 5:**
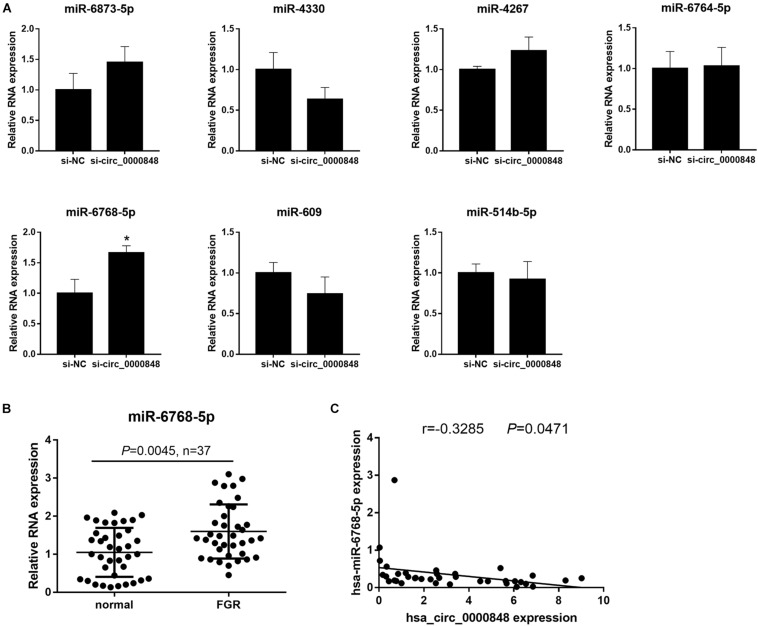
hsa-miR-6768-5p expression is up-regulated in FGR placenta and is negatively correlated with hsa_circ_0000848. **(A)** Expression of hsa-miR-609, hsa-miR-514b-5p, hsa-miR-6768-5p, hsa-miR-6873-5p, hsa-miR-4330, hsa-miR-4267, and hsa-miR-6764-5p in HTR-8 cells transfected with siRNA targeting hsa_circ_0000848 (si-circ_0000848) or negative control siRNA (si-NC). *P < 0.05 using unpaired *t*-tests. **(B)** hsa-miR-6768-5p expression in FGR or normal placental tissues (*n* = 37). Differences between FGR and normal groups were analyzed using non-parametric tests. **(C)** Linear regression analysis to analyze the correlation of hsa_circ_0000848 and hsa-miR-6768-5p in FGR placental tissues (*n* = 37).

**FIGURE 6:**
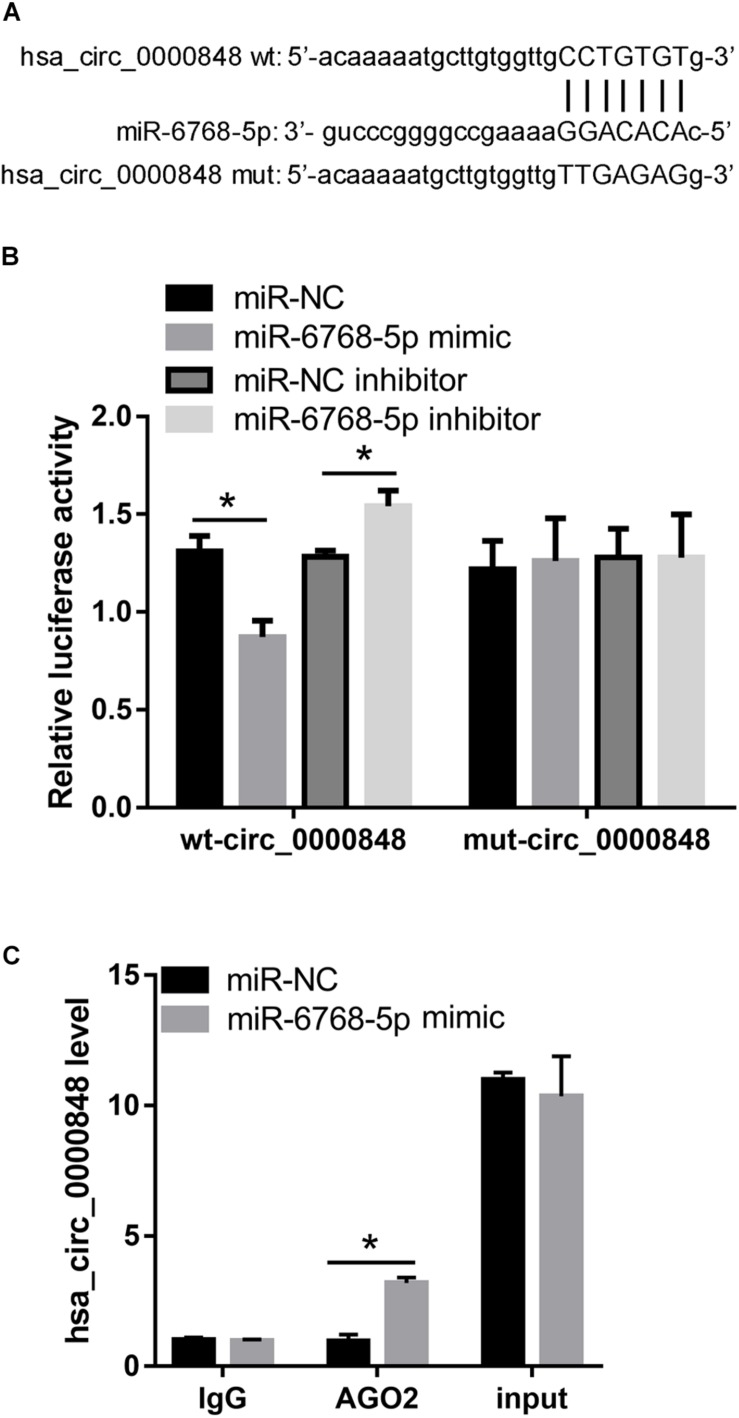
hsa-miR-6768-5p can bind to hsa_circ_0000848 in HTR-8 cells. **(A)** The binding site of hsa-miR-6768-5p on hsa_circ_0000848 and the mutant information for hsa_circ_0000848 in the dual luciferase activity assay. **(B)** Results of the dual luciferase activity assay. **(C)** Results of the anti-AGO2 immunoprecipitation (RIP) assay. hsa_circ_0000848 levels in purified RNA collected from products of anti-AGO2 or anti-IgG RIP assay and cell lysate before immunoprecipitation (input) was detected by RT-qPCR. *P < 0.05 using unpaired *t*-tests. wt, wild type; mut, mutant.

We first investigated the levels of the seven miRNAs in si-circ_0000848 and si-NC HTR-8 cells. We found that only hsa-miR-6768-5p expression was significantly higher in the si-circ_0000848 group than in the si-NC group (*P* = 0.0116) (Figure 5A), indicating that hsa_circ_0000848 knockdown increased hsa-miR-6768-5p levels. Then, hsa-miR-6768-5p expression was further verified in 37 FGR and 37 normal placental tissues. The results indicated that hsa-miR-6768-5p expression was significantly higher in the FGR placental tissues (*n* = 37) than in the normal placental tissues (*n* = 37) (Figure 5B). Linear regression analysis results also yielded a significant negative correlation between hsa-miR-6768-5p and hsa_circ_0000848 levels (*r* = −0.3285, *P* = 0.0471) (Figure 5C). Moreover, a dual luciferase activity assay was carried out to confirm the binding of hsa-miR-6768-5p to linear hsa_circ_0000848 cloned into psi-CHECK2. The binding site of hsa-miR-6768-5p on hsa_circ_0000848 and the effect of the mutant hsa_circ_0000848 on the dual luciferase activity assay are shown in Figure 6A. The dual luciferase activity assay results showed that relative luciferase activity was lower after hsa-miR-6768-5p mimic transfection than after NC transfection, while relative luciferase activity was higher after hsa-miR-6768-5p inhibitor transfection than after miR-NC inhibitor transfection (Figure 6B), indicating that hsa-miR-6768-5p mimic bound to the sequence of hsa_circ_0000848 cloned into psi-CHECK2. Finally, an anti-AGO2 RIP assay was carried out to further investigate whether hsa-miR-6768-5p binds to hsa_circ_0000848. Since AGO2 binds to all miRNAs, the hsa-miR-6768-5p mimic was transfected into HTR-8 cells to increase the percentage of the hsa-miR-6768-5p-AGO2 complex. RIP assay results showed that hsa_circ_0000848 levels were higher in the RIP product of miR-6768-5p-transfected cells than those in the miR-NC-transfected cells (Figure 6C), indicating that hsa-miR-6768-5p bound to endogenous hsa_circ_0000848 in HTR-8 cells.

### Hsa-miR-6768-5p Overexpression Eliminated the Effect of hsa_circ_0000848 Overexpression in HTR-8 Cells

**FIGURE 7:**
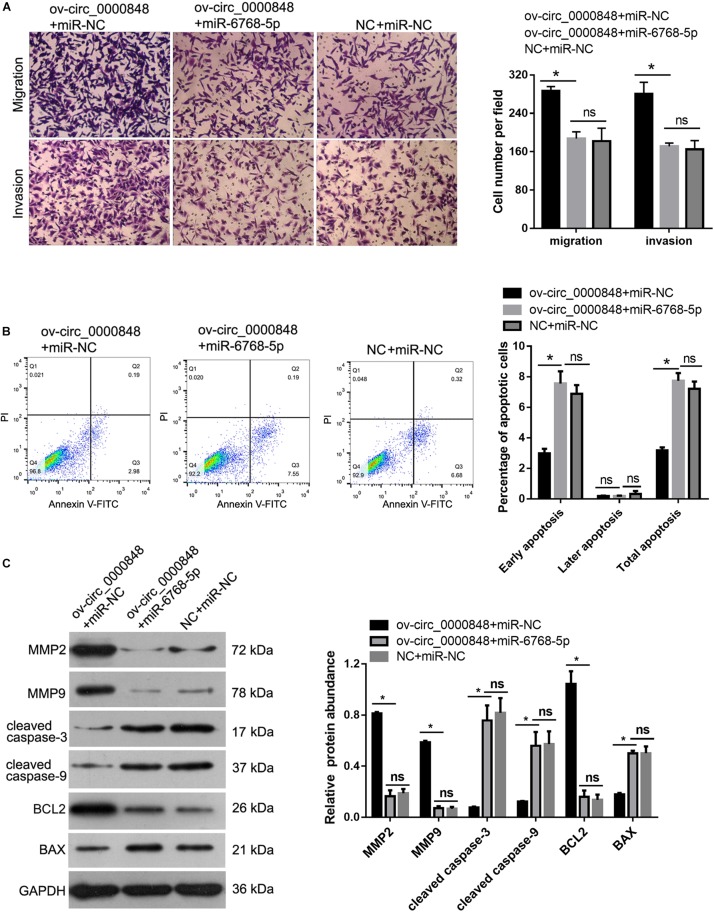
hsa-miR-6768-5p overexpression eliminated the effect of hsa_circ_0000848 overexpression in HTR-8 cells. To confirm whether hsa_circ_0000848 acts by sponging hsa-miR-6768-5p, hsa_circ_0000848 overexpressing plasmid and hsa-miR-6768-5p mimics were co-transfected into HTR-8 cells to carry out rescue assays. Cells transfected with NC plus miR-NC or ov-circ_0000848 + miR-NC were used as controls. The results showed that hsa-miR-6768-5p overexpression eliminated the effect of hsa_circ_0000848 overexpression on migration, invasion **(A)**, apoptosis **(B)** and protein abundance of MMP-2, MMP-9, cleaved caspase-3, cleaved caspase-9, BCL2 and BAX **(C)** in HTR-8 cells. *P < 0.05 using unpaired *t*-tests.

To further confirm whether hsa_circ_0000848 acts by sponging hsa-miR-6768-5p, hsa_circ_0000848 overexpression plasmid and hsa-miR-6768-5p mimics were co-transfected into HTR-8 cells. Cells transfected with NC plus miR-NC or ov-circ_0000848 + miR-NC were used as controls. As shown in Figure 7A, the number of cells that migrated to, or invaded, the bottom chamber was lower in the ov-circ_0000848 + miR-6768-5p group than in the ov-circ_0000848 + miR-NC group. As shown in Figure 7B, the percentage of apoptotic cells was clearly higher in the ov-circ_0000848 + miR-6768-5p group than in the ov-circ_0000848 + miR-NC group. Moreover, the protein abundance of MMP-2, MMP-9, cleaved caspase-3, cleaved caspase-9, BCL2, and BAX was determined. The results showed that the abundance of BCL2, MMP-2, and MMP-9 was lower in the ov-circ_0000848 + miR-6768-5p group than in the ov-circ_0000848 + miR-NC group (Figure 7C). The protein abundance of cleaved caspase-3, cleaved caspase-9, and BAX was higher in the ov-circ_0000848 + miR-6768-5p group than in the ov-circ_0000848 + miR-NC group (Figure 7C). Furthermore, we found that the number of migrated or invaded cells, the percentage of apoptotic cells, and the protein abundance of MMP-2, MMP-9, cleaved caspase-3, cleaved caspase-9, BCL2, and BAX were not significantly different between the ov-circ_0000848 + miR-6768-5p group and NC + miR-NC group (Figure 7), indicating that overexpressing miR-6768-5p and hsa_circ_0000848 simultaneously did not induce obvious phenotypic changes in HTR-8 cells. All these results indicate that hsa-miR-6768-5p overexpression eliminated the effect of hsa_circ_0000848 overexpression in HTR-8 cells.

### Hsa-miR-6768-5p Inhibitor Suppressed Apoptosis and Promoted Migration and Invasion of HTR-8 Cells

**FIGURE 8:**
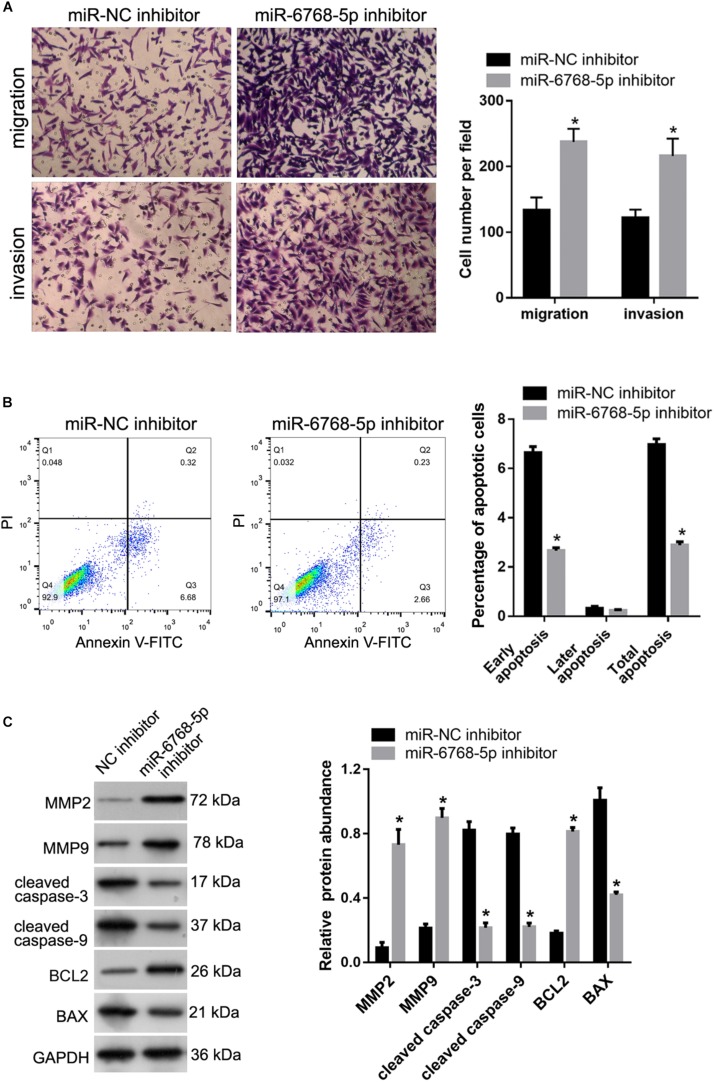
Effect of hsa-miR-6768-5p knockdown on apoptosis, migration and invasion of HTR-8 cells. miR-6768-5p inhibitor was transfected into HTR-8 cells to knockdown hsa-miR-6768-5p. miR-NC inhibitor was transfected as a control. **(A)** The results of the Transwell assay. The left panel includes representative images of migration and invasion by the Transwell assay. The right panel includes bar graphs of the quantification of migrated or invaded cell numbers. **(B)** Effect of hsa-miR-6768-5p knockdown on apoptosis. The left panel shows quantification of the percentage of apoptotic cells. The right panel shows representative graphs of the apoptosis detected using a flow cytometer. **(C)** Effect of hsa-miR-6768-5p knockdown on protein abundance of MMP-2, MMP-9, cleaved caspase-3, cleaved caspase-9, BCL2, and BAX in HTR-8 cells. The left panel includes representative images of western blots. The right panel graphs represent the quantification of relative protein expression levels. *P < 0.05 unpaired *t*-tests.

To evaluate the functional role of hsa-miR-6768-5p in FGR, miR-NC inhibitor and miR-6768-5p inhibitor were transfected into HTR-8 cells. We investigated the role of hsa-miR-6768-5p in the migration and invasion of HTR-8 cells by performing a Transwell assay. The results showed that the number of cells that migrated to, or invaded, the bottom chamber was higher in the miR-6768-5p inhibitor group than in the miR-NC inhibitor group (Figure 8A). We subsequently characterized the role of hsa-miR-6768-5p in HTR-8 cell apoptosis. As shown in Figure 8B, the percentage of apoptotic miR-6768-5p inhibitor group cells was clearly lower than in the miR-NC inhibitor group. The protein abundance of MMP-2, MMP-9, cleaved caspase-3, cleaved caspase-9, BCL2, and BAX was also determined. The abundance of BCL2, MMP-2, and MMP-9 was higher in the miR-6768-5p inhibitor group than in the miR-NC inhibitor group (Figure 8C). Furthermore, the protein abundance of cleaved caspase-3, cleaved caspase-9, and BAX was lower in the miR-6768-5p inhibitor group than in the miR-NC inhibitor group (Figure 8C).

### Predicted Targets, Signaling Pathways and Biological Processes of hsa-miR-6768-5p

To preliminarily explore the mechanisms of hsa-miR-6768-5p, we aimed to predict the target mRNAs. As shown in Supplementary Table S3, 8,137 target mRNAs were predicted. Base on the results of GO and KEGG analysis, we found that 482 targets of hsa-miR-6768-5p were implicated in pathways involved in proliferation, apoptosis and metastasis (Supplementary Table S4). Importantly, we identified SMAD7 as one potential target of hsa-miR-6768-5p. According to the information in circBase, hsa_circ_0000848 is located in an intron of the SMAD7 gene.

## Discussion

FGR is a major cause of perinatal morbidity (Nardozza et al., 2017), although the mechanisms are not fully understood. Thus, a better understanding of the underlying molecular mechanisms of FGR progression is needed. CircRNAs are highly conserved and widely expressed in mammalian cells (Rybak-Wolf et al., 2015). Unlike miRNAs and long-non-coding RNAs, circRNAs are more stable and less degradable with a covalently closed loop structure (Qu et al., 2015). In view of these advantages, circRNAs have great potential as ideal biomarkers for the diagnosis of diseases. Nevertheless, little is known about the role of circRNAs in FGR placenta. Therefore, it is important to obtain a comprehensive view of the circRNA expression profile and function to understand the etiology and pathogenesis of FGR.

The placenta plays a decisive role in fetal growth by connecting the mother with the fetus. Therefore, we first examined circRNA expression profiles in three placentas of FGR fetuses. After validating the expression of three candidate circRNAs in additional samples, hsa_circ_0000848 was confirmed to be significantly down-regulated in FGR tissues. The functional role of hsa_circ_0000848 in FGR was evaluated by investigating its role in migration, invasion, and apoptosis of HTR-8 cells. Our results showed that hsa_circ_0000848 overexpression inhibited apoptosis and promoted cell migration and invasion. In addition, we found that hsa_circ_0000848 overexpression increased the abundance of the anti-apoptotic protein BCL2 (Wu et al., 2018) and decreased the abundance of the pro-apoptotic proteins (Wu et al., 2018) cleaved caspase-3, cleaved caspase-9, and BAX in HTR-8 cells. Furthermore, hsa_circ_0000848 overexpression increased the expression levels of MMP2 and MMP9, which promote cell migration and invasion processes (Zhang et al., 2017). Knockdown of hsa_circ_0000848 had the opposite effect to that of hsa_circ_0000848 overexpression. These results indicated that hsa_circ_0000848 may promote survival and metastasis of HTR-8 cells. HTR-8 is a cell line derived from EVT, a subset of placental cells. EVT plays a critical role in fetal growth and survival. EVT cells migrate to, and invade, the uterine wall leading to remodeling of the maternal vasculature (Lyall et al., 2013; Mifsud and Sebire, 2014; Burton and Jauniaux, 2018). Inadequate invasion and migration of EVT cells is one of the triggers of FGR (Mifsud and Sebire, 2014; Zong et al., 2017). Therefore, we hypothesize that hsa_circ_0000848 plays a role in the etiology and pathogenesis of FGR and may be a potential therapeutic target for FGR. However, our hypothesis needs to be confirmed by further *in vivo* evidence.

Increasing evidence shows that circRNAs are involved in the regulation of gene expression by sequestering miRNAs (Patop and Kadener, 2018; Liu et al., 2019). CircRNAs bind to miRNAs and function as “miRNA sponges.” The inhibitory effect of miRNAs on the target genes then disappears, and the expression of the target gene increases. This process is referred to as the competitive endogenous RNA (ceRNA) mechanism (Hansen et al., 2013; Liu et al., 2019). We predicted potential targets of hsa_circ_0000848 via bioinformatic analysis to explore the mechanism of its function. We identified hsa-miR-6768-5p as a potential target of hsa_circ_0000848. Further, we identified a significant increase in hsa-miR-6768-5p expression and a negative correlation between hsa_circ_0000848 and hsa-miR-6768-5p expression levels in FGR placental tissues. Moreover, hsa_circ_0000848 knockdown increased hsa-miR-6768-5p expression in HTR-8 cells. These results indicate a close association between hsa_circ_0000848 and hsa-miR-6768-5p. The association between hsa_circ_0000848 and hsa-miR-6768-5p was further verified by luciferase reporter and AGO2 RIP assays. Luciferase reporter assay results revealed that hsa-miR-6768-5p bound to hsa_circ_0000848. Additionally, the RIP assay indicated that hsa-miR-6768-5p interacted with circ_0000848. Taken together, these results suggest that hsa_circ_0000848 acted as a sponge to hsa-miR-6768-5p and that hsa-miR-6768-5p was a direct target of hsa_circ_0000848. Moreover, we found that hsa-miR-6768-5p overexpression eliminated the effect of hsa_circ_0000848 overexpression in HTR-8 cells. This result indicated that hsa-miR-6768-5p was also involved in the regulation of apoptosis, migration, and invasion of HTR-8 cells, and hsa_circ_0000848 played its role, at least partially, by sponging hsa-miR-6768-5p.

MiRNAs inhibit target mRNA translation or participate in the degradation of target mRNA (Mohr and Mott, 2015; Wehbe et al., 2019). We found that there are several potential targets of the hsa_circ_0000848/hsa-miR-6768-5p axis, and complex pathways are involved in apoptosis and metastasis. Therefore, the identification of the hsa_circ_0000848/hsa-miR-6768-5p axis targets is likely a complicated task. However, we found that SMAD7 was one potential target of hsa-miR-6768-5p, meanwhile hsa_circ_0000848 is located in an intron of the SMAD7 gene. SMAD7 has been reported to antagonize TGF-beta signaling in the placenta and plays a role in trophoblast growth and differentiation (Xu et al., 2016). It is reported that inhibition of TGF-β signaling may be potentially therapeutic for FGR (Alejandre Alcazar et al., 2011; Alcazar et al., 2014). Therefore, we intend to systematically investigate the hsa_circ_0000848/hsa-miR-6768-5p/SMAD7 axis in a future study.

Our study has some limitations. First, the function of hsa_circ_0000848 was evaluated in the EVT cell line, while its differential expression was identified in samples from term placenta after birth, which is devoid of EVT cells. Therefore, there is a logical gap in examining the function of hsa_circ_0000848 in EVT cells. The experiment was designed this way because there is no way to ethically obtain a developing placenta with trophoblast cells for research. Second, we did not carry out in-depth exploration of other mechanisms that might regulate hsa-miR-6768-5p and hsa_circ_0000848 besides the ceRNA mechanism. Our luciferase reporter assay results and RIP assay did prove that hsa_circ_0000848 could sponge hsa-miR-6768-5p, but RT-qPCR also demonstrated a reduction in hsa -miR-6768-5p after hsa_circ_0000848 overexpression, and upregulation of hsa-miR-6768-5p after hsa_circ_0000848 knockdown. Therefore, we hypothesized that there are other mechanisms that regulate hsa-miR-6768-5p by hsa_circ_0000848. Finally, a small number of human samples were used in the microarray analysis, leading to results that the FDRs of the differentially expressed circRNAs are >0.05.

In conclusion, hsa_circ_0000848 expression was significantly down-regulated in FGR placental tissues. Further, hsa_circ_0000848 also promoted trophoblast cell migration and invasion, and inhibited cell apoptosis via sponging hsa-miR-6768-5p. Based on the importance of the role of EVT in FGR (Mifsud and Sebire, 2014; Zong et al., 2017), we hypothesize that hsa_circ_0000848 plays a role in the etiology and pathogenesis of FGR. Our results provide new avenues for targeted FGR therapy.

## Data Availability Statement

The datasets GENERATED for this study can be found in the NCBI GEO https://www.ncbi.nlm.nih.gov/geo/query/acc.cgi?acc=GSE147721.

## Ethics Statement

The studies involving human participants were reviewed and approved by Ethics Committee of Shenzhen Maternity and Child Health Hospital, Shenzhen, China. The patients/participants provided their written informed consent to participate in this study.

## Author Contributions

HW, JX, and JL contributed to the conception and design of the study. HW wrote the first version of the manuscript. HW, JZ, and ZX performed the experiments. JY, YX, YL, and BL constructed the figures. JY and YX were involved in the clinical data analysis. All authors contributed to revising the manuscript, and read and approved the submitted version.

## Conflict of Interest

The authors declare that the research was conducted in the absence of any commercial or financial relationships that could be construed as a potential conflict of interest.

## Supplementary Material

FIGURE S1 The list of miRNAs that may be potential targets of hsa_circ_0000848.

TABLE S1 Detailed characteristics of participants.

TABLE S2 Sequences of primers used in this study.

TABLE S3 Predicted targets of hsa-miR-6768-5p.

TABLE S4 Predicted targets of hsa-miR-6768-5p implicated in pathways involved in proliferation, apoptosis and metastasis.

## References

[B1] Agarwal V. Bell G. W. Nam J. W. Bartel D. P. (2015). Predicting effective microRNA target sites in mammalian mRNAs. *eLife* 4:e5005. 10.7554/eLife.05005 26267216 PMC4532895

[B2] Alcazar M. A. Dinger K. Rother E. Ostreicher I. Vohlen C. Plank C. (2014). Prevention of early postnatal hyperalimentation protects against activation of transforming growth factor-beta/bone morphogenetic protein and interleukin-6 signaling in rat lungs after intrauterine growth restriction. *J. Nutr.* 144 1943–1951. 10.3945/jn.114.197657 25411031

[B3] Alejandre Alcazar M. A. Morty R. E. Lendzian L. Vohlen C. Oestreicher I. Plank C. (2011). Inhibition of TGF-beta signaling and decreased apoptosis in IUGR-associated lung disease in rats. *PLoS One* 6:e26371. 10.1371/journal.pone.0026371 22028866 PMC3197638

[B4] Benjamini Y. Drai D. Elmer G. Kafkafi N. Golani I. (2001). Controlling the false discovery rate in behavior genetics research. *Behav. Brain Res.* 125 279–284. 10.1016/s0166-4328(01)00297-2 11682119

[B5] Burton G. J. Jauniaux E. (2018). Pathophysiology of placental-derived fetal growth restriction. *Am. J. Obstet. Gynecol.* 218 S745–S761. 10.1016/j.ajog.2017.11.577 29422210

[B6] Deng Q. Chen Y. Yin N. Shan N. Luo X. Tong C. (2015). N-acetylglucosaminyltransferase V inhibits the invasion of trophoblast cells by attenuating MMP2/9 activity in early human pregnancy. *Placenta* 36 1291–1299. 10.1016/j.placenta.2015.08.014 26349781

[B7] Figueras F. Gratacos E. (2017). An integrated approach to fetal growth restriction. *Best Pract. Res. Clin. Obstet. Gynaecol.* 38 48–58. 10.1016/j.bpobgyn.2016.10.006 27940123

[B8] Gynecologists A. C. O. O. A. (2013). ACOG Practice bulletin no. 134: fetal growth restriction. *Obstet. Gynecol.* 121 1122–1133. 10.1097/01.AOG.0000429658.85846.f9 23635765

[B9] Hansen T. B. Jensen T. I. Clausen B. H. Bramsen J. B. Finsen B. Damgaard C. K. (2013). Natural RNA circles function as efficient microRNA sponges. *Nature* 495 384–388. 10.1038/nature11993 23446346

[B10] Hsiao K. Y. Sun H. S. Tsai S. J. (2017). Circular RNA - New member of noncoding RNA with novel functions. *Exp. Biol. Med.* 242 1136–1141. 10.1177/1535370217708978 28485684 PMC5478007

[B11] Huppertz B. Hemmings D. Renaud S. J. Bulmer J. N. Dash P. Chamley L. W. (2005). Extravillous trophoblast apoptosis–a workshop report. *Placenta* 26(Suppl. A), S46–S48. 15837067 10.1016/j.placenta.2005.02.002

[B12] Kristensen L. S. Andersen M. S. Stagsted L. V. W. Ebbesen K. K. Hansen T. B. Kjems J. (2019). The biogenesis, biology and characterization of circular RNAs. *Nat. Rev. Genet.* 20 675–691. 10.1038/s41576-019-0158-7 31395983

[B13] Lasda E. Parker R. (2014). Circular RNAs: diversity of form and function. *RNA* 20 1829–1842. 10.1261/rna.047126.114 25404635 PMC4238349

[B14] Liu L. Yang X. Li N. F. Lin L. Luo H. (2019). Circ_0015756 promotes proliferation, invasion and migration by microRNA-7-dependent inhibition of FAK in hepatocellular carcinoma. *Cell Cycle* 18 2939–2953. 10.1080/15384101.2019.1664223 31522588 PMC6791692

[B15] Lyall F. Robson S. C. Bulmer J. N. (2013). Spiral artery remodeling and trophoblast invasion in preeclampsia and fetal growth restriction: relationship to clinical outcome. *Hypertension* 62 1046–1054. 10.1161/HYPERTENSIONAHA.113.01892 24060885

[B16] Mifsud W. Sebire N. J. (2014). Placental pathology in early-onset and late-onset fetal growth restriction. *Fetal Diagn. Ther.* 36 117–128. 10.1159/000359969 24577279

[B17] Mohr A. M. Mott J. L. (2015). Overview of microRNA biology. *Semin. Liver Dis.* 35 3–11. 10.1055/s-0034-1397344 25632930 PMC4797991

[B18] Nardozza L. M. Caetano A. C. Zamarian A. C. Mazzola J. B. Silva C. P. Marcal V. M. (2017). Fetal growth restriction: current knowledge. *Arch. Gynecol. Obstet.* 295 1061–1077. 10.1007/s00404-017-4341-9 28285426

[B19] Patop I. L. Kadener S. (2018). circRNAs in cancer. *Curr. Opin. Genet. Dev.* 48 121–127. 10.1016/j.gde.2017.11.007 29245064 PMC5877416

[B20] Qu S. Yang X. Li X. Wang J. Gao Y. Shang R. (2015). Circular RNA: a new star of noncoding RNAs. *Cancer Lett.* 365 141–148. 10.1016/j.canlet.2015.06.003 26052092

[B21] Reister F. Frank H. G. Kingdom J. C. Heyl W. Kaufmann P. Rath W. (2001). Macrophage-induced apoptosis limits endovascular trophoblast invasion in the uterine wall of preeclamptic women. *Lab. Invest.* 81 1143–1152. 10.1038/labinvest.3780326 11502865

[B22] Robb K. P. Cotechini T. Allaire C. Sperou A. Graham C. H. (2017). Inflammation-induced fetal growth restriction in rats is associated with increased placental HIF-1alpha accumulation. *PLoS One* 12:e0175805. 10.1371/journal.pone.0175805 28423052 PMC5397034

[B23] Rybak-Wolf A. Stottmeister C. Glazar P. Jens M. Pino N. Giusti S. (2015). Circular RNAs in the mammalian brain are highly abundant. conserved, and dynamically expressed. *Mol. Cell* 58 870–885. 10.1016/j.molcel.2015.03.027 25921068

[B24] Salzman J. (2016). Circular RNA expression: its potential regulation and function. *Trends Genet.* 32 309–316. 10.1016/j.tig.2016.03.002 27050930 PMC4948998

[B25] Sharma D. Farahbakhsh N. Shastri S. Sharma P. (2016a). Intrauterine growth restriction - part 2. *J. Matern. Fetal. Neonatal. Med.* 29 4037–4048. 26979578 10.3109/14767058.2016.1154525

[B26] Sharma D. Shastri S. Farahbakhsh N. Sharma P. (2016b). Intrauterine growth restriction - part 1. *J. Matern. Fetal. Neonatal. Med.* 29 3977–3987. 26856409 10.3109/14767058.2016.1152249

[B27] Shen L. Zhang S. Li Q. Fu Y. Tang G. Jiang Y. (2018). The landscape of non-coding RNA in an adult pig model of intrauterine growth restriction. *Cell Physiol. Biochem.* 50 1764–1778. 10.1159/000494794 30384377

[B28] Shen X. Y. Zheng L. L. Huang J. Kong H. F. Chang Y. J. Wang F. (2019). CircTRNC18 inhibits trophoblast cell migration and epithelial-mesenchymal transition by regulating miR-762/Grhl2 pathway in pre-eclampsia. *RNA Biol.* 16 1565–1573. 10.1080/15476286.2019.1644591 31354028 PMC6779405

[B29] Wehbe N. Nasser S. A. Pintus G. Badran A. Eid A. H. Baydoun E. (2019). MicroRNAs in cardiac hypertrophy. *Int. J. Mol. Sci.* 20:4714. 10.3390/ijms20194714 31547607 PMC6801828

[B30] Wu H. Medeiros L. J. Young K. H. (2018). Apoptosis signaling and BCL-2 pathways provide opportunities for novel targeted therapeutic strategies in hematologic malignances. *Blood Rev.* 32 8–28. 10.1016/j.blre.2017.08.004 28802908

[B31] Xu J. Sivasubramaniyam T. Yinon Y. Tagliaferro A. Ray J. Nevo O. (2016). Aberrant TGFbeta Signaling Contributes to Altered Trophoblast Differentiation in Preeclampsia. *Endocrinology* 157 883–899. 10.1210/en.2015-1696 26653761

[B32] Yu T. Wang Y. Fan Y. Fang N. Wang T. Xu T. (2019). CircRNAs in cancer metabolism: a review. *J. Hematol. Oncol.* 12:90. 10.1186/s13045-019-0776-8 31484561 PMC6727394

[B33] Zhang C. Wang L. Chen J. Liang J. Xu Y. Li Z. (2017). Knockdown of Diaph1 expression inhibits migration and decreases the expression of MMP2 and MMP9 in human glioma cells. *Biomed. Pharmacother.* 96 596–602. 10.1016/j.biopha.2017.10.031 29035824

[B34] Zhou W. Wang H. Yang J. Long W. Zhang B. Liu J. (2019). Down-regulated circPAPPA suppresses the proliferation and invasion of trophoblast cells via the miR-384/STAT3 pathway. *Biosci. Rep.* 39:BSR20191965. 10.1042/BSR20191965 31427481 PMC6732364

[B35] Zong L. Wei X. Gou W. Huang P. Lv Y. (2017). Zinc improves learning and memory abilities of fetal growth restriction rats and promotes trophoblast cell invasion and migration via enhancing STAT3-MMP-2/9 axis activity. *Oncotarget* 8 115190–115201. 10.18632/oncotarget.23122 29383152 PMC5777764

