# Supplementary figures and images for: Circular RNA hsa_circ_0000848 Promotes Trophoblast Cell Migration and Invasion and Inhibits Cell Apoptosis by Sponging hsa-miR-6768-5p
Source: Front Cell Dev Biol. 2020 May 19;8:278. doi: 10.3389/fcell.2020.00278 (PMC7249963; doi:10.3389/fcell.2020.00278)

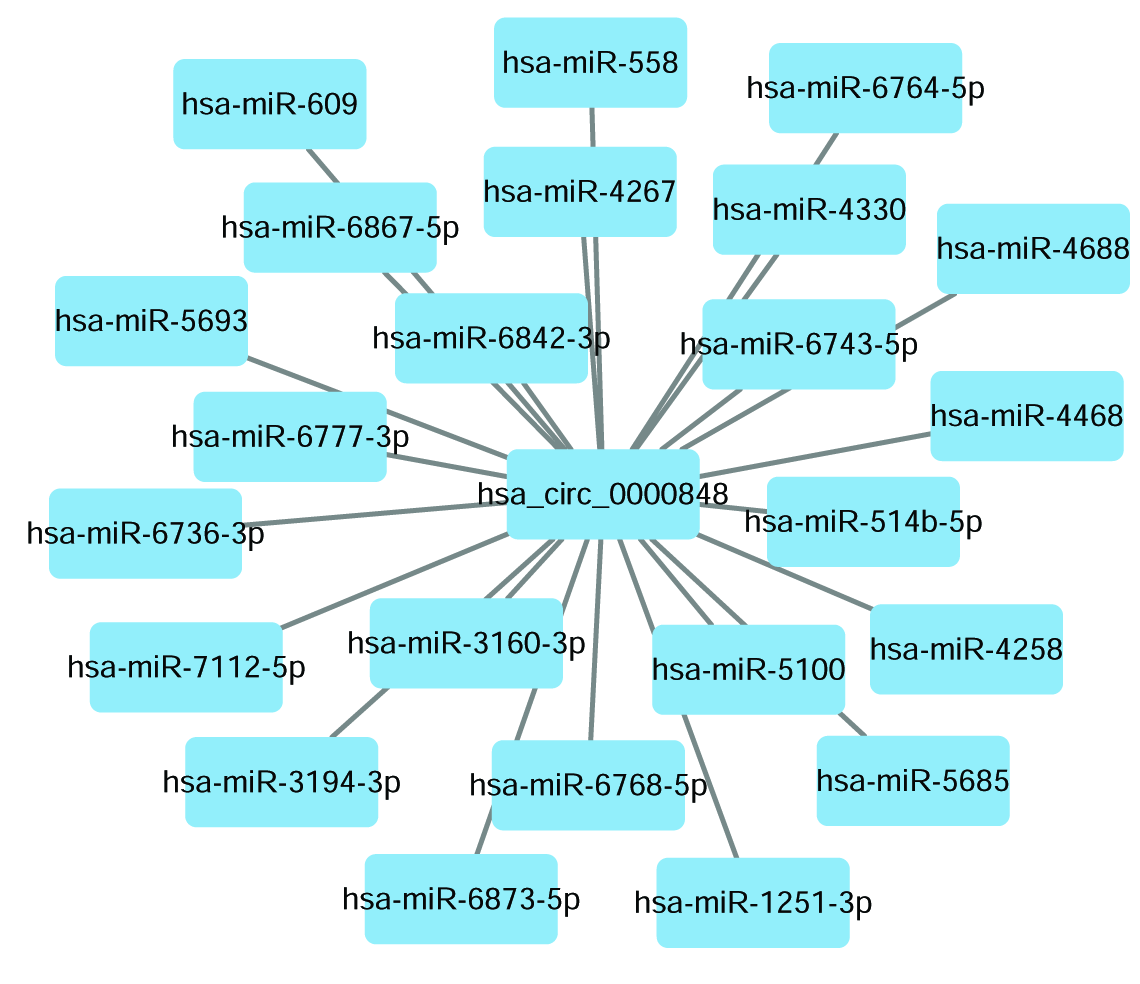

Supplement: FIGURE S1 — The list of miRNAs that may be potential targets of hsa_circ_0000848. [file Image_1.TIF]
