# Supplementary material for: Circular RNA hsa_circ_0000848 Promotes Trophoblast Cell Migration and Invasion and Inhibits Cell Apoptosis by Sponging hsa-miR-6768-5p
Source: Front Cell Dev Biol. 2020 May 19;8:278. doi: 10.3389/fcell.2020.00278 (PMC7249963; doi:10.3389/fcell.2020.00278)
Supplement: TABLE S2 — Sequences of primers used in this study. [file Table_2.DOCX]

Supplementary Table 2 Sequences of primers used in this study

| **Primer name** | **Primer sequence (5’-3’)** |
| --- | --- |
| hsa_circ_0007738-F | CTGGGTTGCATGGACCTTTA |
| hsa_circ_0007738-R | AGGGCCGGTCTTTTCTTCTAA |
| hsa_circ_0071271-F | GCACTCTATGTGCTTTCATTC |
| hsa_circ_0071271-R | CCACTCCAGCTCCTCTTCTT |
| hsa_circ_0000848-F | CTCAGCTCTGCTCTGAGATG |
| hsa_circ_0000848-R | TTAATTAAGCCTCCACCCCC |
| miR-6873-5p-F | acactccagctgggcagagggaatacagagggc |
| miR-6873-5p-RT | ctcaactggtgtcgtggagtcggcaattcagttgagattgccctc |
| miR-4330-F | acactccagctgggcctcagatcagagcct |
| miR-4330-RT | ctcaactggtgtcgtggagtcggcaattcagttgaggcaaggct |
| miR-4267-F | acactccagctgggaccacctcccctgcaaacgt |
| miR-4267-RT | acactccagctgggtccagctcggtgg |
| miR-6764-5p-F | acactccagctgggtcccagggtctggtcagat |
| miR-6764-5p-RT | ctcaactggtgtcgtggagtcggcaattcagttgagcaactctga |
| miR-6768-5p-F | acactccagctgggcacacaggaaaagcggggc |
| miR-6768-5p-RT | ctcaactggtgtcgtggagtcggcaattcagttgagcagggccccg |
| miR-609-F | acactccagctgggagggtgtttctctcatc |
| miR-609-RT | ctcaactggtgtcgtggagtcggcaattcagttgagagagatga |
| miR-514b-5p-F | acactccagctgggttctcaagagggaggcaa |
| miR-514b-5p-RT | ctcaactggtgtcgtggagtcggcaattcagttgagatgattgcc |
| U6-F | CTCGCTTCGGCAGCACA |
| U6-R | AACGCTTCACGAATTTGCGT |
| U6-RT | AACGCTTCACGAATTTGCGT |
| GAPDH-R | GAGTGGGTGTCGCTGTTGA |
